# No difference in renal injury and fibrosis between wild-type and NOD1/NOD2 double knockout mice with chronic kidney disease induced by ureteral obstruction

**DOI:** 10.1186/s12882-018-0867-8

**Published:** 2018-04-02

**Authors:** Ingrid Stroo, Diba Emal, Loes M. Butter, Gwen J. Teske, Nike Claessen, Mark C. Dessing, Stephen E. Girardin, Sandrine Florquin, Jaklien C. Leemans

**Affiliations:** 1 Department of Pathology, Academic Medical Center, University of Amsterdam, Meibergdreef 9, room L2-112, 1105 AZ Amsterdam, The Netherlands; 2 Department of Pathology, Radboud University Nijmegen Medical Center, Nijmegen, The Netherlands; 3 Department of Laboratory Medicine and Pathobiology, University of Toronto, Toronto, Canada

**Keywords:** Pattern recognition receptors, NOD1, NOD2, Renal fibrosis, Obstructive nephropathy

## Abstract

**Background:**

Chronic kidney disease (CKD) is characterized by sustained tissue damage and ongoing tubulo-interstitial inflammation and fibrosis. Pattern recognition receptors (PRRs) including Toll-like receptors (TLRs) and NOD-like receptors (NLRs) can sense endogenous ligands released upon tissue damage, leading to sterile inflammation and eventually irreversible kidney disease. It is known that NOD1 and NOD2 contribute to the pathogenesis of various inflammatory diseases, including acute kidney injury. However their role in chronic kidney disease is largely unknown. The aim of this study was therefore to investigate the contribution of NOD1 and NOD2 in renal interstitial fibrosis and obstructive nephropathy.

**Methods:**

To do so, we performed unilateral ureteral obstruction (UUO) in wild type (WT) and NOD1/NOD2 double deficient (DKO) mice and analysed renal damage, fibrosis and inflammation. Data were analysed using the non-parametric Mann-Whitney U-test.

**Results:**

Minor changes in inflammatory response were observed in NOD1/2 DKO mice, while no effects were observed on renal injury and the development of fibrosis.

**Conclusion:**

No difference in renal injury and fibrosis between WT and NOD1/NOD2 DKO mice following obstructive nephropathy induced by ureteral obstruction.

**Electronic supplementary material:**

The online version of this article (10.1186/s12882-018-0867-8) contains supplementary material, which is available to authorized users.

## Background

NOD1 and NOD2 are members of the cytoplasmic PRR family of NLRs. PRRs are important in mediating a rapid response to pathogens via recognition of several highly conserved pathogen- associated molecular patterns (PAMP). In addition to PAMPs various (endogenous) damage-associated molecular patterns (DAMP) or stress signals have been identified that can initiate sterile inflammation [1]. Upon renal injury DAMPs are released such as biglycan, high-mobility group box 1 (HMGB1), and hyaluronic acid that can signal via TLRs and NLRs [2–5]. NOD1 and NOD2 detect specific substructures from bacterial peptidoglycan (PGN). NOD1 senses Gram^−^-derived PGN containing Tri-DAP [6, 7], while NOD2 senses Gram^−^- and Gram^+^-derived PGN containing MDP [8, 9]. In line, we found that NOD1/2 are involved in the development of acute renal disease during septic shock induced by bacterial components [10]. In addition to bacterial structures, Sabbah et al. reported the activation of NOD2 by single-stranded RNA viruses [11]. Recently, activation of NOD1 and NOD2 by the non-pathogenic derived cell permeable small molecule DMXAA was reported [12]. As far as we know, no endogenous DAMPs for NOD1 and NOD2 are described. However, based on their structural and functional similarities with other NLR family members and TLRs it could be speculated that NOD1 and NOD2 are also activated by currently unknown endogenous ligands. In line with this reasoning, Shigeoka et al. showed that NOD1 and NOD2 participate in acute renal ischemia reperfusion injury (IRI), suggesting that these receptors are able to respond to endogenous ligands released upon IRI [13]. NOD1 is widely expressed in many cell types and organs including the tubular epithelial cells (TEC) in human and mouse kidney [13–16]. NOD2 is expressed on murine TECs, mesangial cells, podocytes and on human TECs and glomerular endothelial cells [13, 17, 18]. Given the expression of NOD1 and NOD2 in the kidney and more specific in TEC and the fact that NOD1/2 play a deleterious role in acute kidney disease could suggest that these PRRs contribute to the pathogenesis of chronic renal damage as well. PRRs like NLRP3 and TLR4 have already been shown to play a role in obstructive nephropathy [19–21]. However, nothing is known about the role of NOD1 and NOD2 in inflammation and fibrosis during obstructive nephropathy. In the present study we therefore investigated the role of NOD1 and NOD2 in a model of obstructive nephropathy induced by ureteral obstruction.

## Methods

### Mice

Pathogen-free 8- to 12-week old female C57Bl/6 WT mice were purchased from Janvier (Le Genest, France). NOD1/NOD2 DKO mice were generated from NOD1 and NOD2 knockout mice and backcrossed to C57Bl/6 background at least 10 generations as described before [22]. Previously, we have characterized the DKO mice phenotypically and this revealed that except for lower liver weight in NOD1/2 DKO mice, there were no differences in body/organ weight, leukocyte count/composition and plasma biochemical markers between both strains [10]. NOD1/2 DKO mice (Additional file 1) were bred in the animal facility of the Academic Medical Center in Amsterdam, The Netherlands. Age- and sex-matched mice were used in all experiments. The animal and Use Committee of the University of Amsterdam approved all experiments.

### Unilateral ureter obstruction

Mice (*N* = 9/group) received a pre-operative dose of analgesia (0.15 mg/kg buprenorfine, subcutaneously) and were anesthetized by inhalation of 3% isoflurane, 0.2% N_2_O and 2% O_2_ during the surgical procedure. The right ureter was permanently ligated via a ventral approach using 6–0 silk (Tyco, Gosport, UK). The ureter was ligated at the height of the lower part of the kidney. Mice were sacrificed 3, 7 and 14 days after surgery via a heart puncture (blood collection) followed by cervical dislocation under general anaesthesia. Kidneys were snap frozen in liquid nitrogen and stored at -80 °C or fixed in 10% formalin o/n prior to further processing. Contralateral non-obstructed kidneys served as control.

### Quantitative real-time RT-PCR

Total RNA was extracted from kidney using the TRIzol® reagent (Invitrogen, Breda, The Netherlands) and converted to cDNA. Quantitative real-time RT-PCR was performed on a LightCycler® 480 System (Roche, Mijdrecht, The Netherlands) using LightCycler® 480 SYBR Green I Master mix (Roche). Specific gene expression was normalized towards the reference gene TATA box binding protein (TBP). Primer sequences are as follows: NOD1 forward 5′-tcagactcagcgtcaaccag-3′ and reverse 5′-taaacccaggaacgtcacga-3′, NOD2 forward 5′-gggagatgttggagtggaac-3′ and reverse 5′-agcgaagagcacactcaacc-3′, and TBP forward 5′-ggagaatcatggaccagaaca-3′ and reverse 5′-gatgggaattccaggagtca-3′.

### Histology and immunohistochemistry

Formalin-fixed tissue was embedded in paraffin using standard procedures. Four-μm thick sections were cut and used for all stainings. For examining renal histology, sections were stained with periodic acid-Schiff reagents after diastase digestion (PasD). Injury to tubules was assessed (blinded) by determining the percentage of affected tubules per 10 fields (magnification × 400) semi-quantitatively on a scale from 0 to 4 (0 = 0%, 1 = < 25%, 2 = 25–50%, 3 = 50–75%, and 4 = > 75%) according to the following criteria: tubular dilatation, epithelial simplification, and interstitial expansion in the cortex. For immunohistochemistry, sections were stained with FITC-labelled anti-mouse Ly-6G (Pharmingen, BD Biosciences, Alphen a/d Rijn, The Netherlands), rat anti-mouse F4/80 (Serotec, Oxford, UK), rabbit anti-mouse active caspase-3 (Cell Signaling Technology, Beverly, MA, USA), rabbit anti-human Ki67 (Neomarkers, Fremont, CA, USA), rabbit polyclonal to collagen type I (GeneTex, Irvine, CA, USA), or mouse anti-human αSMA (DAKO, Heverlee, Belgium) to detect granulocytes, macrophages, apoptosis, proliferation, collagen type I, and myofibroblasts respectively. The number of Ly6 positive cells and the number of caspase-3 and Ki67 positive TEC was counted in 10 non-overlapping fields (magnification × 400) in a blinded manner. The percentage of positive staining for F4/80, collagen type I, total collagen and αSMA in obstructed kidneys was analysed using a computer-assisted digital analysis program (Image Pro-plus®, Media Cybernetics). At least 15 visual fields were sampled from the cortex of each kidney (magnification × 20).

### Statistical analyses

All statistical analyses were performed using GraphPad Prism 5 software (San Diego, CA, USA). Data were analysed using the non-parametric Mann-Whitney U-test. Results are expressed as mean ± standard error of the mean (SEM). *P* < 0.05 was considered statistically significant.

## Results

**Fig. 1:**
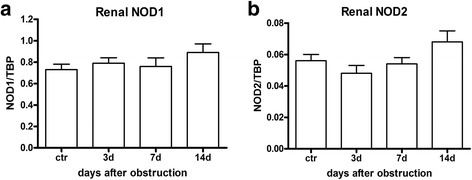
Renal expression of NOD1 (**a**) and NOD2 (**b**) after 0, 3, 7, and 14 days following obstruction. Gene expression was normalized towards the reference gene TBP. Data are expressed as mean ± SEM. *N* = 9/group

**Fig. 2:**
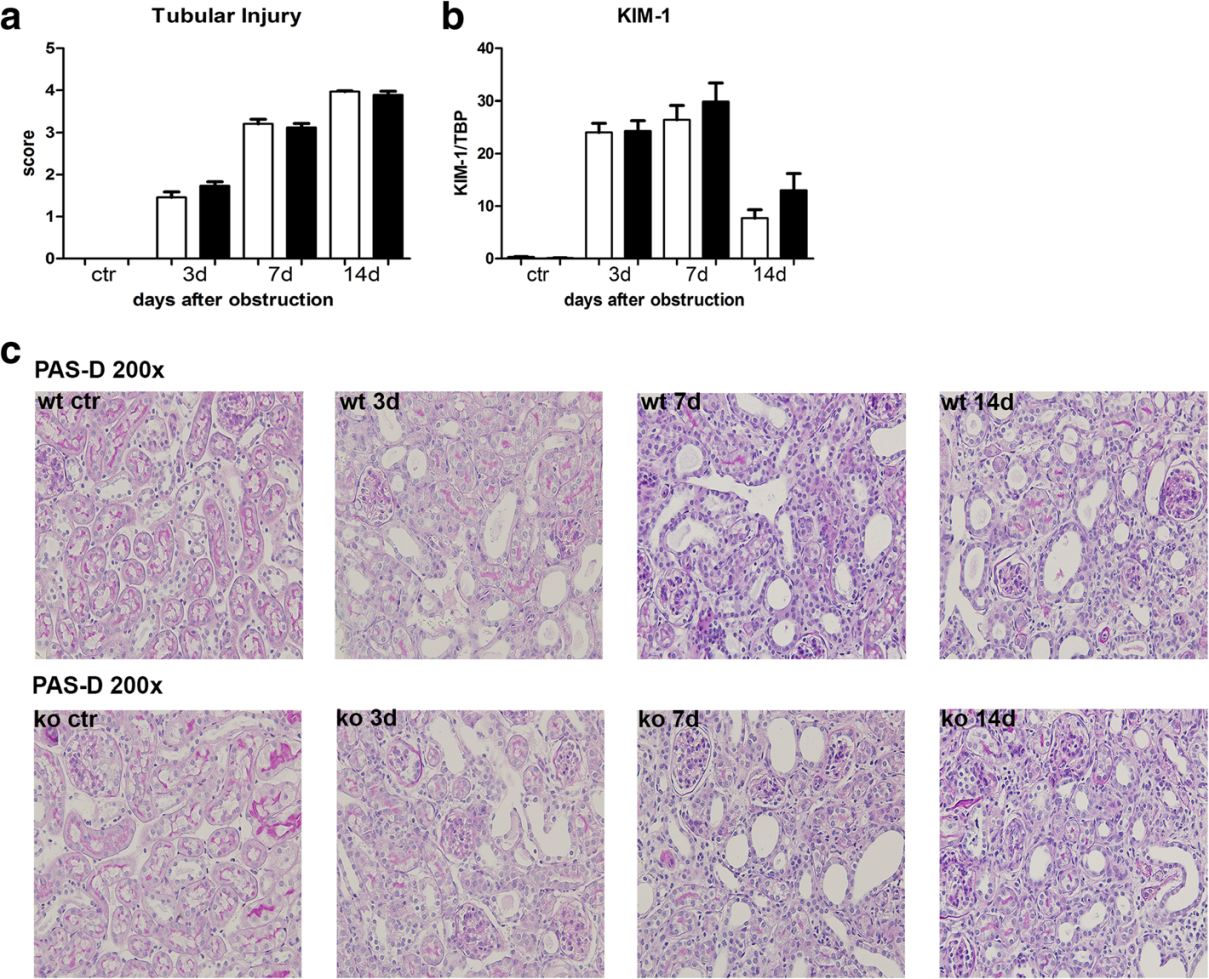
Renal injury in WT (white bars) and NOD1/2 DKO (black bars) mice after 0, 3, 7, and 14 days following obstruction. Renal damage was evaluated by blinded scoring of the necrotic tubules in PAS-D-stained sections (**a**, **c**) and by measuring the mRNA expression of KIM-1 (**b**) in total kidney homogenates. Data are expressed as mean ± SEM. Results were analysed with the non-parametric two-tailed Mann-Whitney U-test. *P < 0.05. *N* = 9/group

**Fig. 3:**
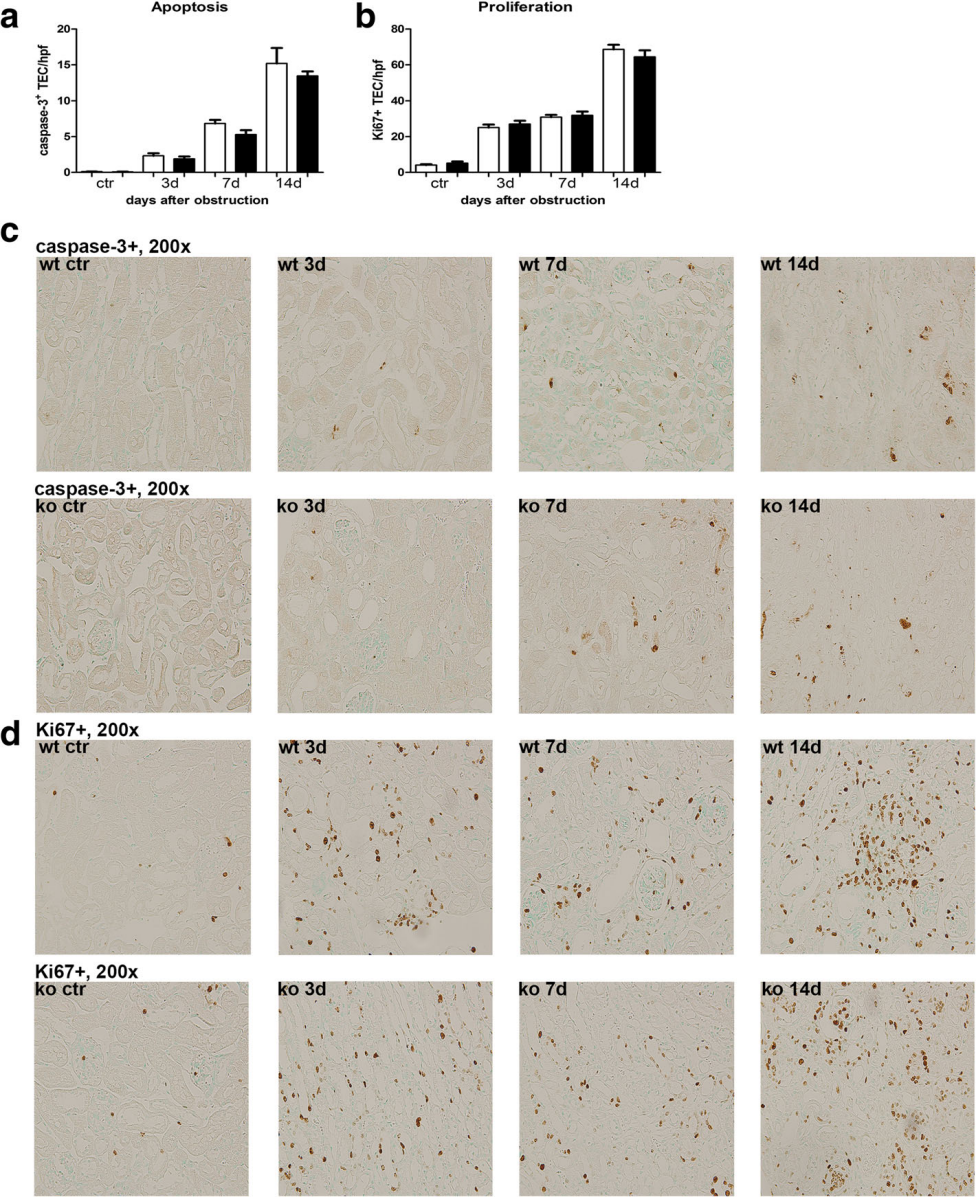
Apoptosis and proliferation of TECs in WT (white bars) and NOD1/2 DKO (black bars) mice after 0, 3, 7, and 14 days following obstruction. The amount of apoptotic TEC was determined by scoring caspase-3+ TEC (**a**, **c**) and the amount of proliferating TEC (**b**, **d**) was determined by scoring Ki67+ TEC. Data are expressed as mean ± SEM. *N* = 9/group

**Fig. 4:**
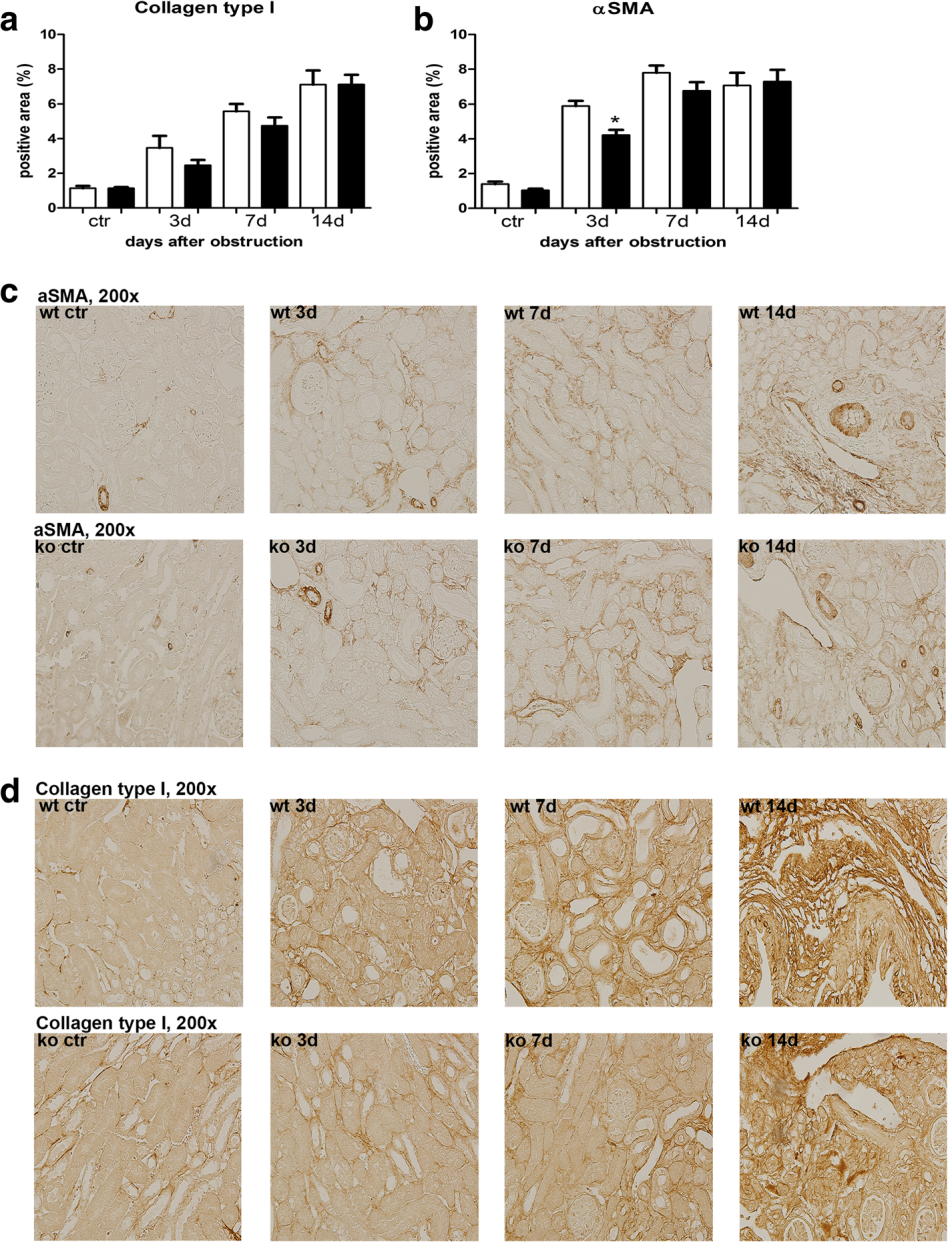
Renal fibrosis in WT (white bars) and NOD1/2 DKO (black bars) mice after 0, 3, 7, and 14 days following obstruction*.* Collagen type I deposition in kidneys was determined by Collagen type I staining and digitally analysed (**a**, **d**). Myofibroblast accumulation in kidneys was assessed by αSMA staining and digitally analysed (**b**, **c**). Data are expressed as mean ± SEM. Results were analysed with the non-parametric two-tailed Mann-Whitney U-test. *P < 0.05. *N* = 9/group

**Fig. 5:**
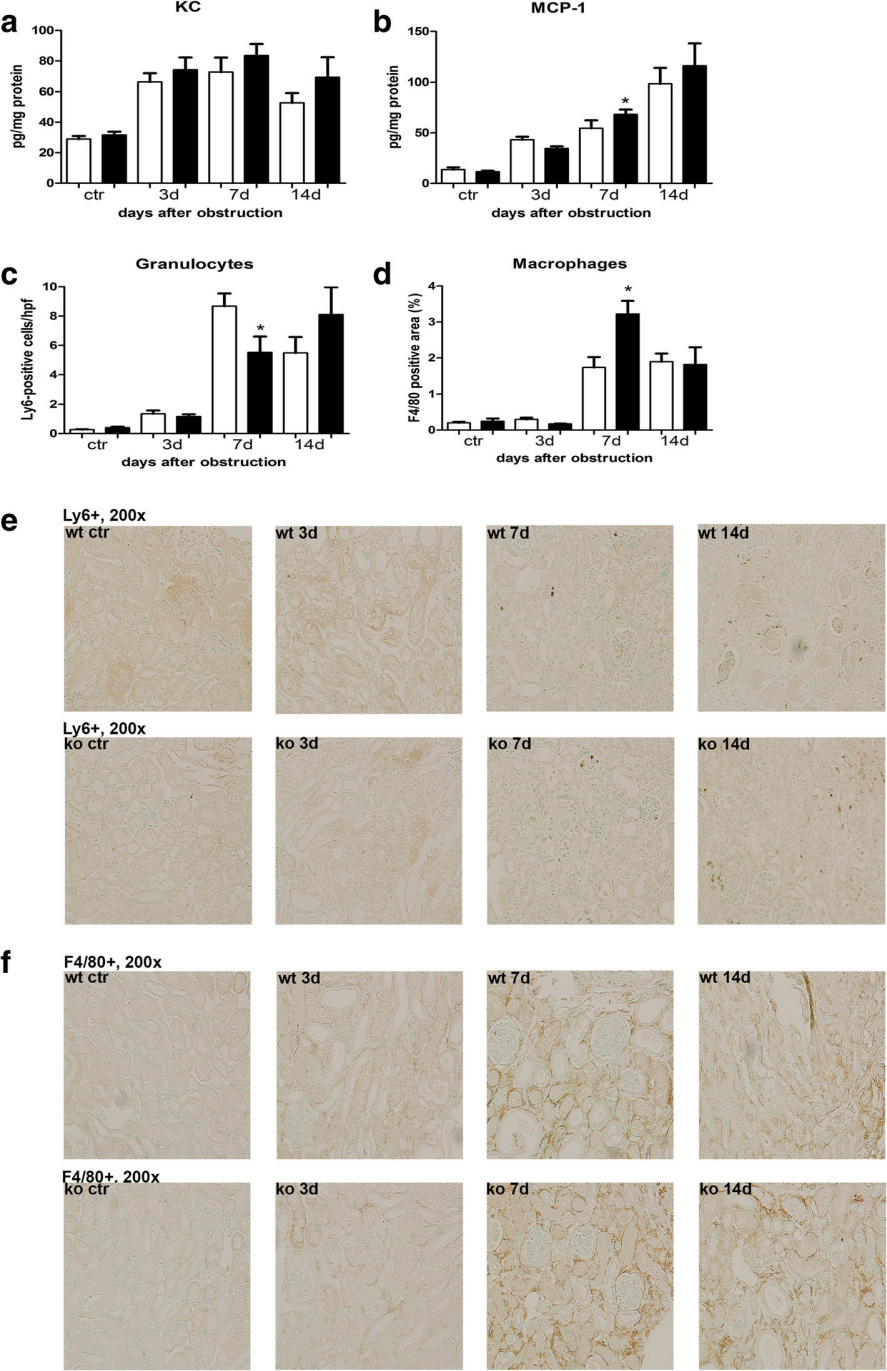
Renal inflammation in WT (white bars) and NOD1/2 DKO (black bars) mice after 0, 3, 7, and 14 days following obstruction*.* KC (**a**) and MCP-1 (**b**) were measured in total kidney homogenates with specific ELISAs. Influx of granulocytes was determined by scoring Ly6+ cells (**c**, **e**). Influx of macrophages was assessed by F4/80 staining which was digitally analysed (**d**, **f**). Data are expressed as mean ± SEM. Results were analysed with the non-parametric two-tailed Mann-Whitney U-test. *P < 0.05. *N* = 9/group

The role of NOD1 and NOD2 in CKD was investigated using the mouse model UUO. First we analysed the expression of NOD1 and NOD2 mRNA in WT kidney at several time points after UUO. We found expression of both transcripts in the kidney (Fig. 1), which were not altered during the development of obstructive nephropathy. Tubular injury, as assessed by scoring PAS-D-stained kidney sections, increased markedly after UUO with a similar degree of damage in WT and NOD1/2 DKO at all time points examined (Fig. 2a, c). In line with the injury score, there were no differences in KIM-1 expression between the WT and the KO mice at all time points (Fig. 2b). KIM-1 was declined at day 14 of UUO possibly as an adaptation to prevent or slow down KIM-1 mediated chronic inflammation and renal fibrosis, as described previously [23]. Tubulointerstitial injury in obstructed kidneys can result in an imbalance between TEC apoptosis and proliferation. Apoptosis and proliferation of TEC was increased at all investigated time points after obstruction (Fig. 3). However, no difference between WT and NOD1/2 DKO mice was observed. Fibrosis was determined by collagen type I (Fig. 4a) and total collagen deposition (Additional file 2: Figure S2). In both WT and NOD1/2 DKO obstructed kidneys fibrosis increased progressively, however no difference between the WT and NOD1/2 DKO mice was observed. Next we analysed the amount of myofibroblasts by αSMA immunohistochemistry (Fig. 4b). In line with tubular injury and fibrosis, the amount of myofibroblasts increased after UUO. Although myofibroblast accumulation was lower in NOD1/2 DKO mice 3 days following ureteral obstruction compared to WT mice, no differences were found after 7 and 14 days (Fig. 4b). One of the early events in progressive renal injury is the induction of chemokines and the subsequent recruitment of inflammatory cells. The granulocyte chemoattractant KC (Fig. 5a) and the monocyte chemoattractant MCP-1 (Fig. 5b) increased significantly following obstruction in both WT and NOD1/2 DKO kidneys. Except for a slight but significant higher MCP-1 level in kidneys from NOD1/2 DKO mice compared with WT 7 days following obstruction, no difference in KC and MCP-1 levels were observed between the WT and NOD1/2 DKO mice. The influx of granulocytes (Fig. 5c, e) and accumulation of macrophages (Fig. 5d, f) increased in the obstructed WT and NOD1/2 DKO kidneys, yet there were no differences in these parameters between the WT and NOD1/2 DKO mice, 3 and 14 days post UUO. Seven days following obstruction there was a slight but significant decrease in granulocyte influx and a significant increase in macrophage accumulation in NOD1/2 DKO kidneys compared with WT kidneys. To get more insight in the activation of the NF-kB signaling pathway, we measured the downstream cytokines IL-1b and TNF-α in total kidney homogenates. This revealed no significant differences between the WT and NOD1/2 DKO mice, expect for IL-1b day 3 (Additional file 3: Figure S3). Together these results reveal that there are no or minor changes in the inflammatory response, renal damage and fibrosis following CKD induced by ureteral obstruction in NOD1/2 DKO mice compared with WT mice.

## Discussion

To investigate the role of NOD1 and NOD2 in chronic renal inflammation, injury and fibrosis we subjected NOD1/2 DKO and WT mice at various time points to UUO. UUO initiates a sequence of events in the obstructed kidney, including interstitial inflammation and TEC death, ultimately leading to renal fibrosis which is the final common pathway for numerous forms of progressive renal disease. Recently, the role of the PRRs TLR2, TLR9, TLR4 and NLRP3 in progressive renal injury was investigated. Although TLR2 initiates the inflammatory response during obstructive nephropathy, it does not play a significant role in the development of renal progressive injury and fibrosis [3, 24]. Similarly, TLR9 was not involved in the pathogenesis of UUO [24]. On the other hand, TLR4 attenuates tubular damage and does contribute to renal fibrosis during obstructive nephropathy as demonstrated by an increased injury score and decreased collagen deposition in TLR4-deficient mice [20, 25]. Other work implied a central role for NLRP3 in renal inflammation, fibrosis and tubular damage at different phases of UUO [19, 21]. Apparently, different PRR members have unique response during obstructive nephropathy that lead to a profoundly different outcome of local injury and tubulointerstitial inflammation and fibrosis.

From our study we conclude that NOD1 and NOD2 do not play a significant role in the development of tubulointerstitial fibrosis and inflammation nor in the progression of renal damage after UUO-induced injury. No differences were observed between WT and NOD1/2 DKO obstructed kidneys regarding tubular injury score, apoptosis, proliferation and myofibroblast accumulation. A marginal effect of NOD1/2 deficiency could be detected in the inflammatory response during obstructive nephropathy. Slightly more MCP-1 and concomitant increased macrophage accumulation was observed in the NOD1/2 DKO kidney 7 days following obstruction, while granulocyte influx was lower at this time point. The majority of infiltrating leukocytes into the UUO-damaged kidney are macrophages, which produce cytokines responsible for tubular apoptosis and fibroblast proliferation and activation. However, enhanced macrophage accumulation did not affect the progression of renal fibrosis in NOD1/2 DKO mice. In another study on progressive kidney disease, namely diabetes, it was shown that NOD2 is upregulated and promoted the transcription of extracellular matrix genes and renal injury by inducing inflammation and podocyte insulin resistance [18]. Moreover, in sepsis- and ischemia-induced acute kidney disease models, NOD1/2 DKO mice were demonstrated to be protected against renal disease [10, 13]. Apparently, as it was the case for TLR2 [3, 26], NOD1 and NOD2 are involved in the initiation of inflammation but do not necessarily contribute to further renal damage and fibrosis. Considering that we found no difference between WT and NOD1/2 DKO mice in renal pathology, one could speculate that despite their structural and functional similarities with other PRRs, NOD1 and NOD2 are not activated by DAMPs that are released after UUO. This would be in line with current literature in which solely bacterial ligands and not DAMPs are described to activate NOD1 and NOD2. We anticipate that the difference in renal pathology between WT and NOD1/2DKO mice in the renal ischemia reperfusion injury model might be due to translocation of bacterial products across the leaky intestinal barrier that activate NOD1/2 resulting in inflammation-associated nephropathy. The phenomenon of intestinal barrier disruption is known to occur after renal ischemia reperfusion injury [27] but is not described for UUO.

This study however, has some limitations which have to be pointed out. By using double knockout mice we cannot rule out the possibility that either NOD1 or NOD2 play a different role in UUO and compensate each other. Alternatively, the function of NOD1 or NOD2 might be masked in our knockout model by redundancy or compensatory mechanism. Our experiments tested moreover only unilateral ureteral obstruction with its own advantages and limitations [28] and no other animal models of chronic kidney disease and fibrosis. Other possible roles of NOD1/NOD2 in these disorders that may be activated under different circumstances remain therefore to be tested.

Taken together, our data do not show a functional role for NOD1/2 in kidney injury and fibrosis following chronic kidney disease induced by ureteral obstruction and suggest that similar to infection, different forms of sterile kidney disease will be sensed by different PRRs triggering different signalling pathways which culminate in different kidney disease outcomes.

## Conclusion

Together these results reveal that there are no or minor changes in the inflammatory response, renal damage and fibrosis following obstructive nephropathy induced by ureteral obstruction in NOD1/2 DKO mice compared with WT mice.

## Additional files

Additional file 1: **Figure S1**. The genotype of NOD1/2 DKO mice. Genomic DNA from mice was amplified by PCR with specific primers to detect the disrupted sequences on a 1% agarose gel with a 100 bp marker. First 5 bands are the KO mice and the last bands are the WT mice in A and B. *N* = 5/3 per group. (TIFF 12868 kb)

Additional file 2: **FigureS2.** Total collagen in kidneys of WT (white bars) and NOD1/2 DKO (black bars) mice after 0, 3, 7, and 14 days following obstruction. Total collagen was assessed by Picro Sirius Red staining which was digitally analysed (A, B). Data are expressed as mean ± SEM. *N* = 9/group. (TIFF 1068 kb)

Additional file 3: **Figure S3**. Renal inflammation in WT (white bars) and NOD1/2 DKO (black bars) mice after 0, 3, 7, and 14 days following obstruction. IL-1b (A) and TNF-α (B) were measured in total kidney homogenates with specific ELISAs. Data are expressed as mean ± SEM. Results were analysed with the non-parametric two-tailed Mann-Whitney U-test. *P < 0.05. *N* = 9/group. (TIFF 2728 kb)

## Abbreviations

CKD: Chronic kidney disease
DAMP: Danger associated molecular patterns
DKO: Double deficient
HMGB1: High-mobility group box 1
IRI: Ischemia reperfusion injury
NLRs: NOD-like receptors
PAMP: Pathogen associated molecular patterns
PGN: Peptidoglycan
PRRs: Pattern recognition receptors
SEM: Standard error of the mean
TEC: Tubular epithelial cell
TLRs: Toll-like receptors
UUO: Unilateral ureteral obstruction
WT: Wild type
