# Supplementary figures and images for: No difference in renal injury and fibrosis between wild-type and NOD1/NOD2 double knockout mice with chronic kidney disease induced by ureteral obstruction
Source: BMC Nephrol. 2018 Apr 2;19:78. doi: 10.1186/s12882-018-0867-8 (PMC5879837; doi:10.1186/s12882-018-0867-8)

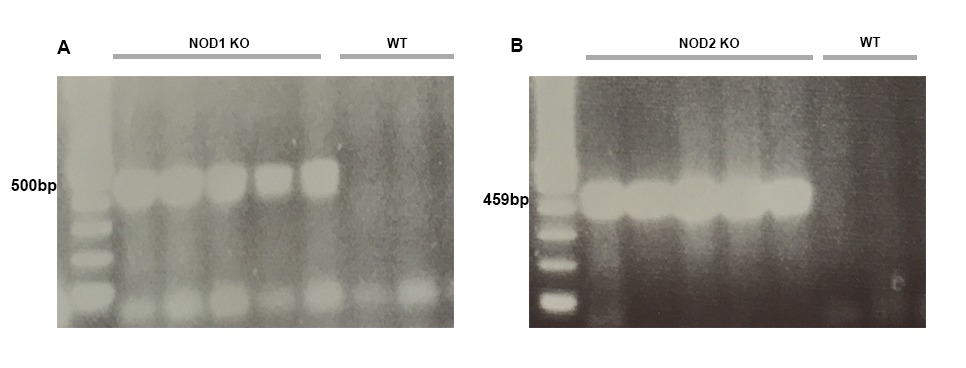

Supplement: Supplementary file 1 — Figure S1. The genotype of NOD1/2 DKO mice. Genomic DNA from mice was amplified by PCR with specific primers to detect the disrupted sequences on a 1% agarose gel with a 100 bp marker. First 5 bands are the KO mice and the last bands are the WT mice in A and B. N = 5/3 per group. (TIFF 12868 kb) [file 12882_2018_867_MOESM1_ESM.tif]

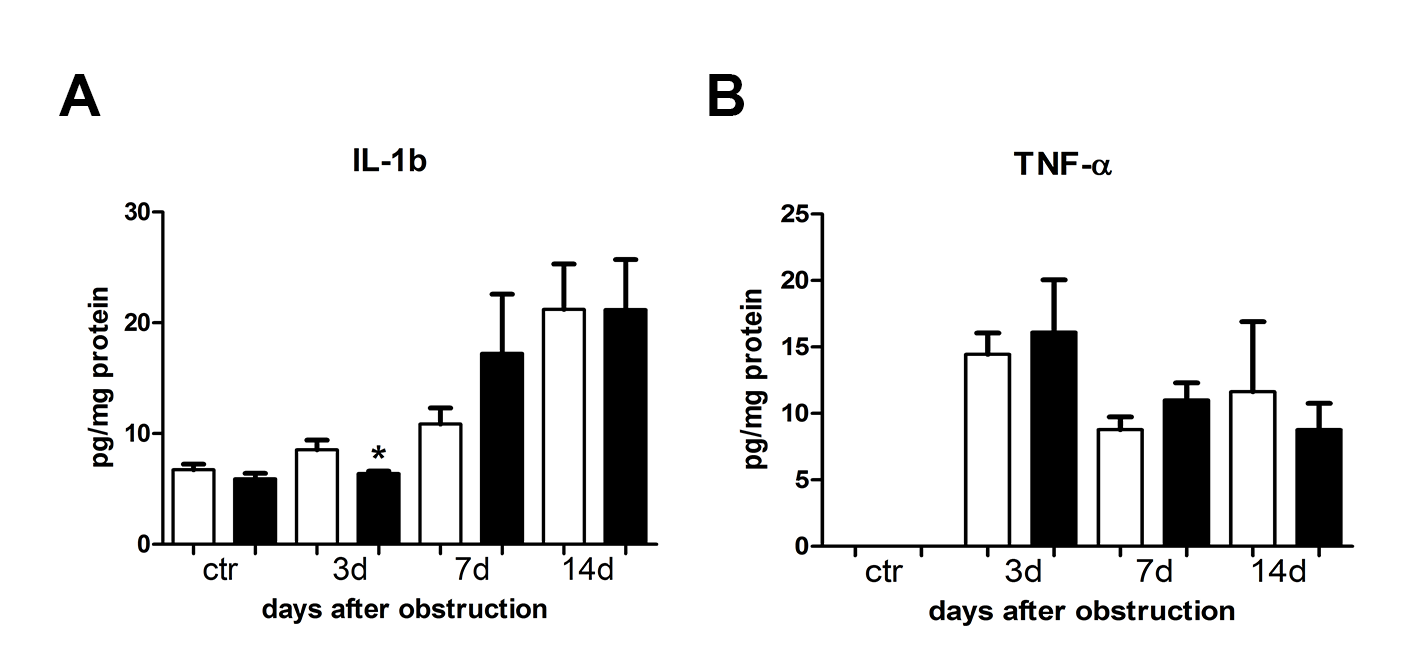

Supplement: Supplementary file 2 — FigureS2. Total collagen in kidneys of WT (white bars) and NOD1/2 DKO (black bars) mice after 0, 3, 7, and 14 days following obstruction. Total collagen was assessed by Picro Sirius Red staining which was digitally analysed (A, B). Data are expressed as mean ± SEM. N = 9/group. (TIFF 1068 kb) [file 12882_2018_867_MOESM3_ESM.tif]

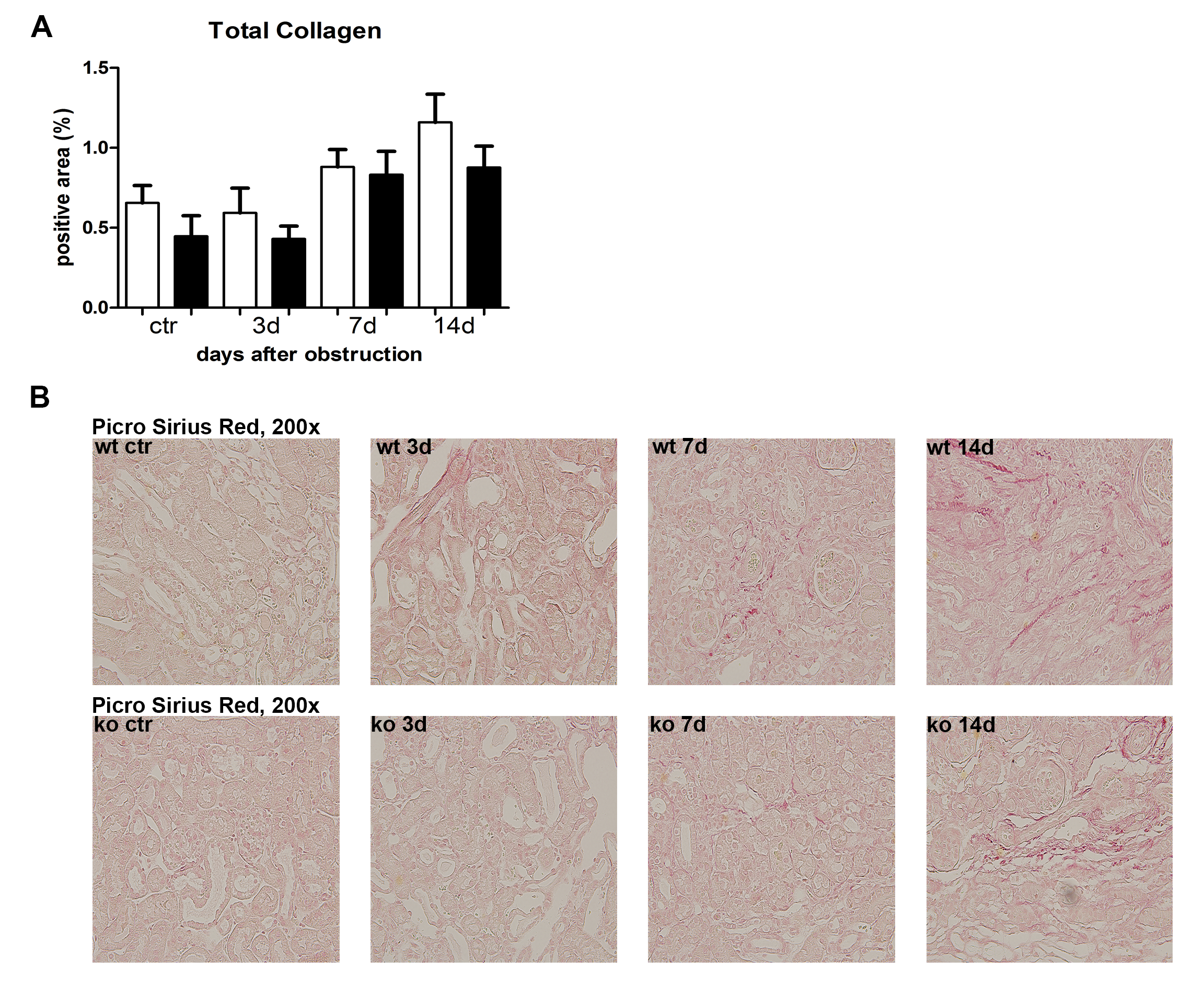

Supplement: Supplementary file 3 — Figure S3. Renal inflammation in WT (white bars) and NOD1/2 DKO (black bars) mice after 0, 3, 7, and 14 days following obstruction. IL-1b (A) and TNF-α (B) were measured in total kidney homogenates with specific ELISAs. Data are expressed as mean ± SEM. Results were analysed with the non-parametric two-tailed Mann-Whitney U-test. *P < 0.05. N = 9/group. (TIFF 2728 kb) [file 12882_2018_867_MOESM2_ESM.tif]
